# Supplementary material for: Dietary lipids fuel GPX4-restricted enteritis resembling Crohn’s disease
Source: Nat Commun. 2020 Apr 14;11:1775. doi: 10.1038/s41467-020-15646-6 (PMC7156516; doi:10.1038/s41467-020-15646-6)
Supplement: Supplementary file 1 — Supplementary Information [file 41467_2020_15646_MOESM1_ESM.pdf]

SUPPLEMENTARY INFORMATION

Dietary lipids fuel GPX4-restricted enteritis resembling Crohn's disease

Mayr et al.

Supplementary Figures

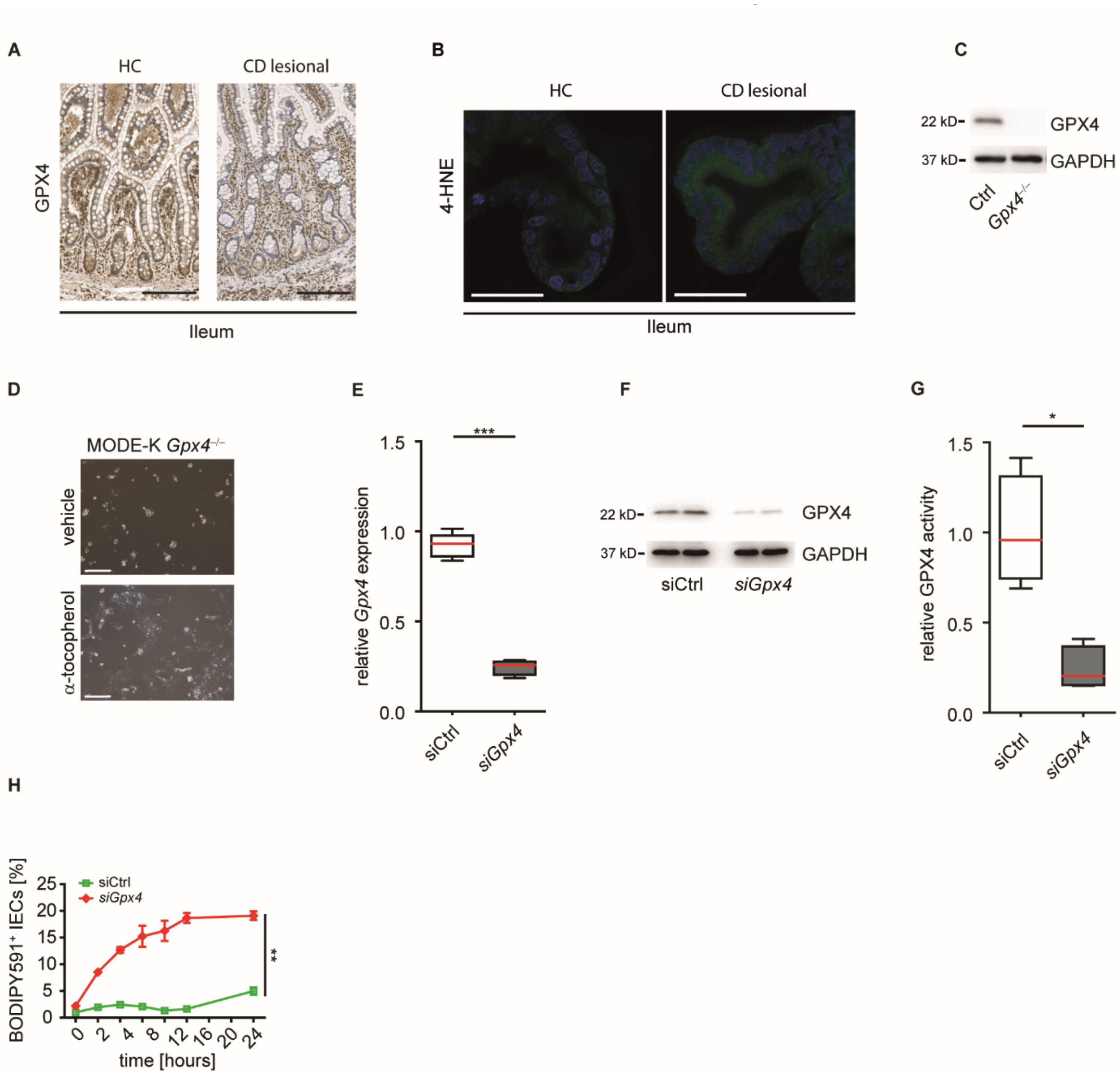

Supplementary Figure 1. GPX4 restricts lipid peroxidation in IECs. (A) Representative GPX4 immunoreactivity (brown) determined in the lesional small intestine of CD patients as compared to HC. (n=7 patients per group). Scale bars indicate 200 µm**. (B)** Representative confocal microscopy images of 4-HNE-labelled human small intestinal organoids of CD patients and HC. (n=2 biologically independent samples). Scale bars indicate 50µm. **(C)** GPX4 quantification by western blot after MODE-K CRISPR Cas9-editing of Exon 1 in the *Gpx4* gene. (n=1). **(D)** Representative light microscopy image of CRISPR Cas9-edited *Gpx4*-/- MODE-K IECs. Note that all *Gpx4*-/- IECs are detached from the culture plate as indicated by their coccus-like shape. In contrast, α-tocopherol treatment protected against detachment of *Gpx4*-/- IECs indicative of some viability. (n=2 biologically independent experiments). Scale bars indicate 25µm. **(E)** Relative *Gpx4* expression of siRNA silenced MODE-K IECs determined by qPCR. (n=5 biologically independent experiments). ***P<0.001. **(F)** Representative GPX4 western blot of si*Gpx4* IECs. GAPDH served as loading control. (n=3 biologically independent experiments). **(G)** Relative GPX4 enzymatic activity of *siGpx4* IECs

assessed with a colorimetric enzymatic test. (n=4 biologically independent experiments). *P=0.0286. **(H)** Relative LPO assessed by flow cytometry of BODIPY581^{+} C11 at indicated time points of AA stimulation in *siGpx4* and siCtrl IECs. (n=3 biologically independent experiments). ***P>0.001. Data presented as mean +/- SEM. For panel **(E)** and **(G)** data are presented as boxblot with median and interquartile range (25th and 75th). The whiskers represent minimal and maximal values. Unpaired two-tailed Students *T*-test for panel **(E)**, Mann Whitney Test for panel **(G)** and two-way ANOVA with Bonferroni's multiple comparison test for panel **(H)**. Source data are provided as a Source Data file.

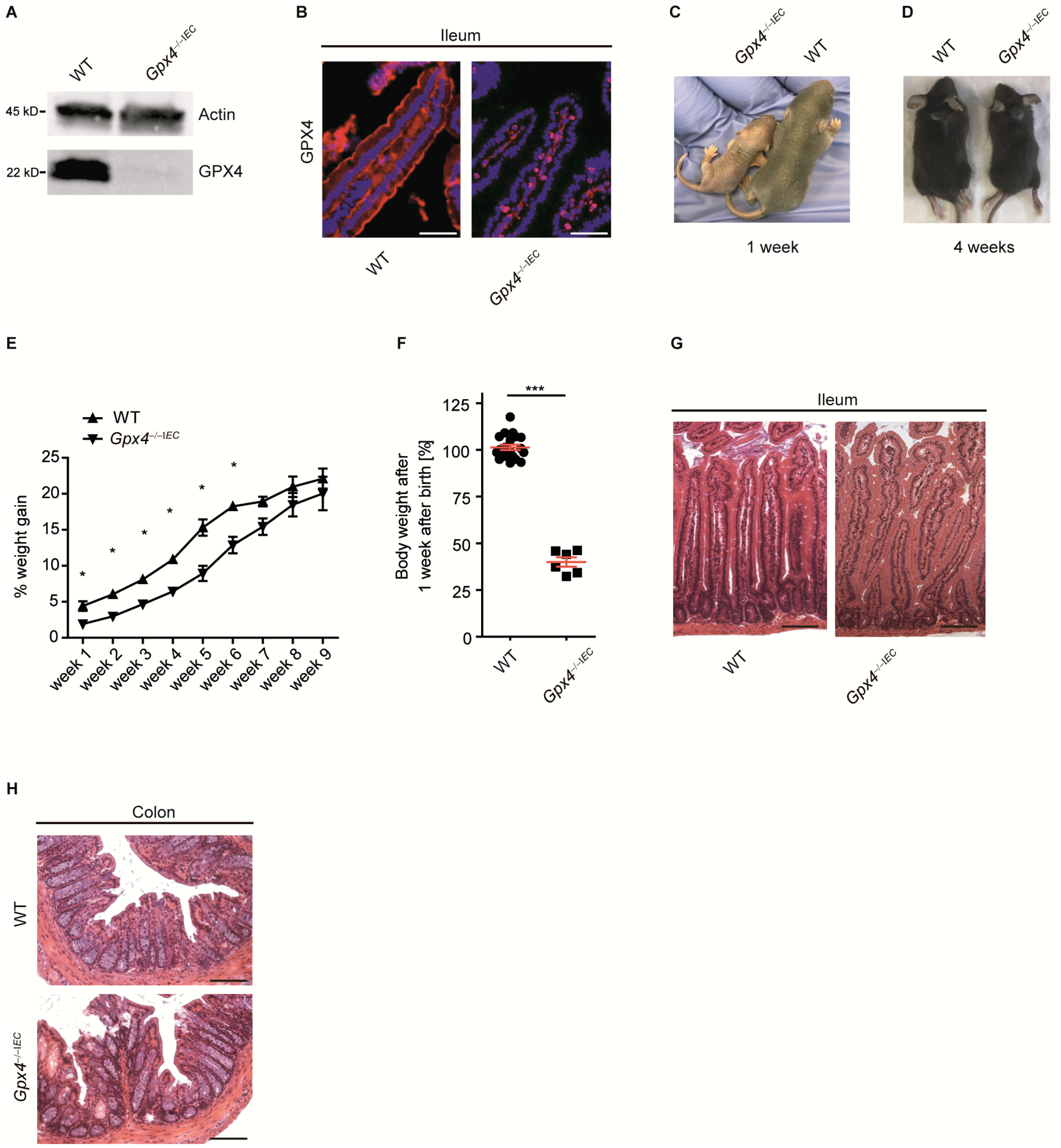

Supplementary Figure 2. α-tocopherol supplementation rescues embryonic lethality of *Gpx4*$^{-/-IEC}$ mice. (A, B) Representative GPX4 immunoblot from isolated IECs **(A)** and representative confocal microscopy images of GPX4 labelled small intestinal sections of indicated genotypes on an α-tocopherol supplemented diet **(B)**. Actin served as loading control in **(A)**. (n=3 mice per group). **(C, D)** Representative images of WT and *Gpx4*$^{-/-IEC}$ mice born to mothers exposed to an α-tocopherol enriched diet until weaning. (n=3 mice per group). **(E)** Weight course of *Gpx4*$^{-/-IEC}$ and WT mice on an α-tocopherol enriched diet. (n=3 mice per group). Note that *Gpx4*$^{-/-IEC}$ mice show a reduced body weight one week after birth which they regained after 7 weeks. Data presented as mean +/- SEM. *P<0.05. **(F)** Body weight at 1 week of age of WT and *Gpx4*$^{-/-IEC}$ mice born to a mother that was exposed to an α-tocopherol enriched diet. Each dot represents an individual animal. (n=21 for WT and n=6 for *Gpx4*$^{-/-IEC}$ mice). Data presented as mean +/- SEM. ***P<0.001. **(G, H)** Representative H&E images of indicated genotypes from the small **(G)** and large intestine **(H)** of WT and *Gpx4*$^{-/-IEC}$ mice. Scale bars

indicate 100µm. (n=3 mice per group). Unpaired two-tailed Students *T*-test. Source data are provided as a Source Data file.

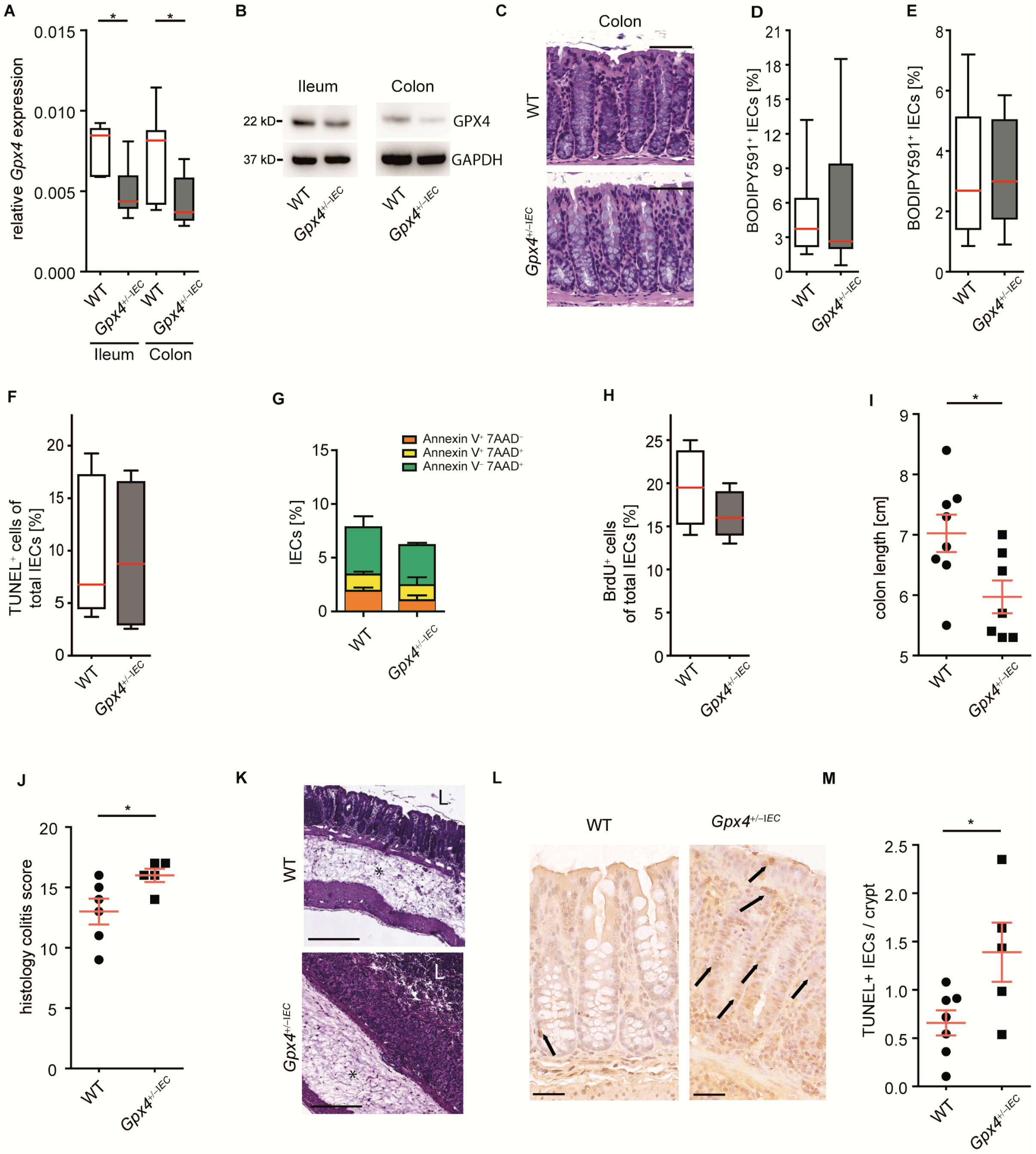

Supplementary Figure 3. ***Gpx4*** $^{+/-IEC}$ **mice are susceptible to DSS-induced colitis. (A)** Relative *Gpx4* expression in small- and large-intestinal epithelial scrapings from indicated genotypes determined by qPCR. (n=6 mice per group). One-way ANOVA with Bonferroni's multiple comparison test. *P=0.0486 (ileum) and *P=0.0270 (colon). **(B)** Representative GPX4 immunoblot of small- and large-intestinal epithelial scrapings from indicated genotypes. GAPDH served as loading control. (n=3 mice per group). **(C)** Representative H&E images of indicated genotypes from the large intestine. Scale bars indicate 100µm. (n=10 mice per group). **(D, E)** Relative LPO assessed by flow cytometry of BODIPY581/591 C11^{+} IECs derived from small intestinal villi **(D)** and crypts **(E)** of *Gpx4*$^{+/-IEC}$ mice as compared to WT. (n=10 WT mice and n=9 *Gpx4*$^{+/-IEC}$ mice). **(F)** Quantification of TUNEL^{+} IECs per crypt in indicated genotypes. (n=5 WT mice and n=4 *Gpx4*$^{+/-IEC}$ mice). **(G)** Quantification of Annexin V^{+} and 7AAD^{+} IECs by flow cytometry of indicated genotypes. (n=8 WT mice and n=6 *Gpx4*$^{+/-IEC}$ mice). **(H)**

Quantification of $BrdU^+$ IECs in indicated genotypes. $BrdU^+$ cells of total IECs along the villus-crypt axis are shown. (n=4 WT mice and n=5 *Gpx4*$^{+/-IEC}$ mice). **(I)** Colon length of indicated genotypes at the closure of the DSS experiment. Each dot represents an experimental animal. (n=8 for WT and n=7 for *Gpx4*$^{+/-IEC}$). *P=0.0252. **(J, K)** Colonic histology score **(J)** of indicated genotypes after DSS treatment with representative H&E images in **(K).** Note the loss of crypt architecture and the protrusion of inflammatory cells into the gut lumen in *Gpx4*$^{+/-IEC}$ mice. L, lumen, asterisk denotes submucosal edema. Scale bars represent 500µm. Each dot represents an experimental animal. (n=7 for WT and n=5 for *Gpx4*$^{+/-IEC}$). *P=0.0433. **(L)** Representative images of TUNEL-labelled sections of indicated genotypes after DSS treatment (arrows denote brown TUNEL positive cells). (n=7 for WT and n=5 for *Gpx4*$^{+/-IEC}$). Scale bar represents 50µm. **(M)** Quantification of $TUNEL^+$ IECs per crypt in indicated genotypes after DSS exposure. (n=7 for WT and n=5 for *Gpx4*$^{+/-IEC}$). *P=0.0339. For panel **(G), (I), (J)** and **(M)** data are presented as mean +/- SEM. For panel **(A), (D-F)** and **(H)** data are presented as boxblot with median and interquartile range (25th and 75th). The whiskers represent minimal and maximal values. For panel **(A)** Kruskal-Wallis test was used, for panel **(D-J)** and **(M)** unpaired two-tailed Students *T*-test was used. Source data are provided as a Source Data file.

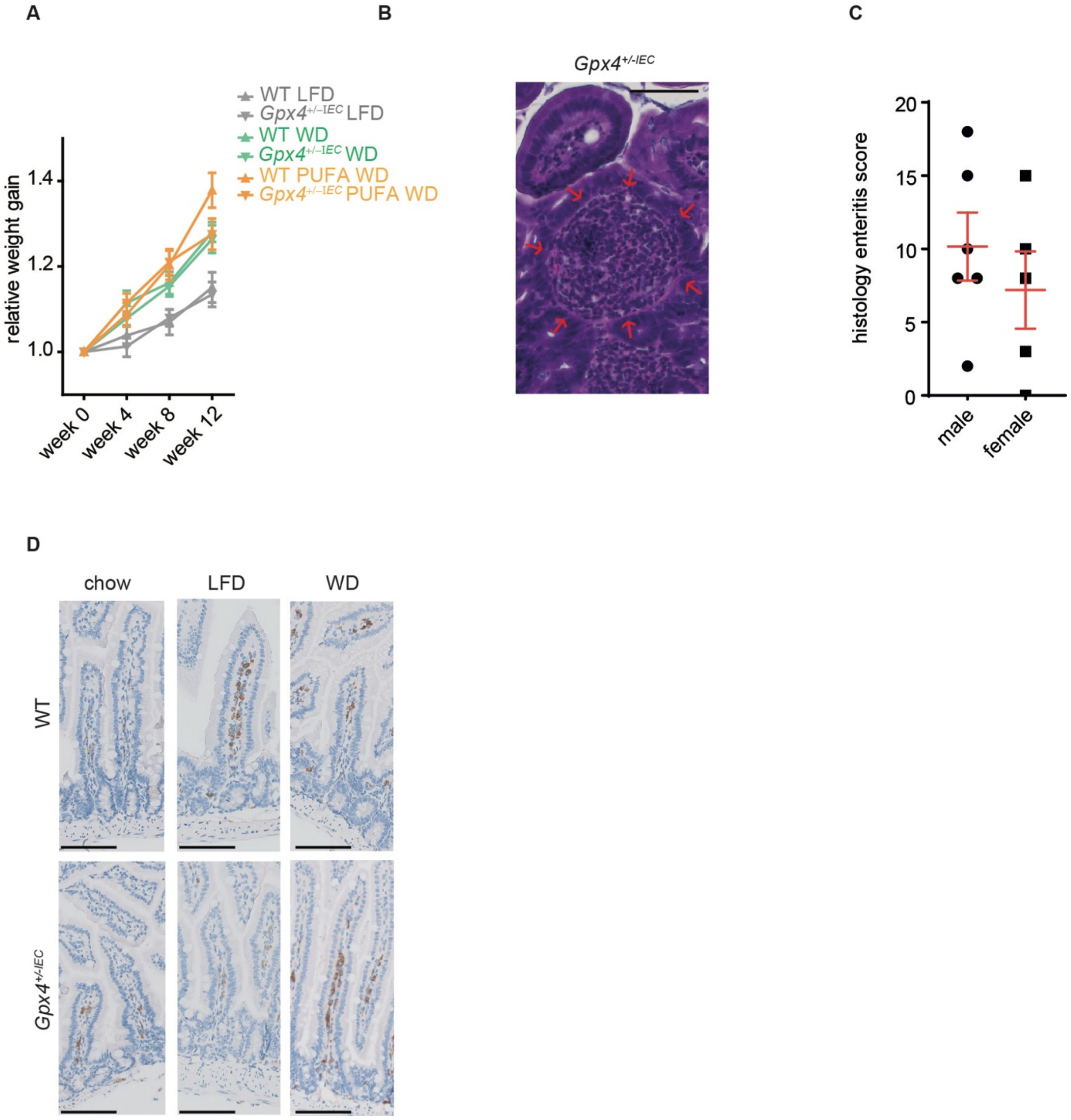

Supplementary Figure 4. *Gpx4*$^{+/-IEC}$ mice develop a focal enteritis on a PUFA-enriched Western diet. (A) Weight course of *Gpx4*$^{+/-IEC}$ and WT mice fed a low-fat diet (LFD), a Western diet (WD) or a PUFA-enriched Western diet (PUFA WD) for 3 months. (n=8 mice for WT LFD, n=9 mice for *Gpx4*$^{+/-IEC}$ LFD, n=7 mice for WT WD, n=10 mice for *Gpx4*$^{+/-IEC}$ WD, n=9 mice for WT PUFA WD and n=11 mice for *Gpx4*$^{+/-IEC}$ PUFA WD). **(B)** Representative H&E image of a granuloma-like lesion (denoted by red arrows) in the small intestine of *Gpx4*$^{+/-IEC}$ mice fed a PUFA WD for 3 months. Scale bar represents 100µm. (n=5 mice per group). **(C)** Histology score of male and female *Gpx4*$^{+/-IEC}$ mice exposed to a PUFA WD for 3 months. Each dot indicates one experimental animal. (n=6 for male *Gpx4*$^{+/-IEC}$ mice and n=5 for female *Gpx4*$^{+/-IEC}$ mice). **(D)** Representative immunohistochemistry images of MPO^{+} cells (brown) in indicated genotypes exposed to a chow diet, a low fat diet (LFD) and a Western diet (WD). (n=4 mice per group). We did not note accumulation of MPO^{+} cells outside the vessels in the villus. Scale bar indicates 100µm. A two-way ANOVA with Bonferroni's multiple comparison

test was used for **(A)**. An Unpaired two-tailed Students *T*-test was used for **(C)**. Data presented as mean +/-SEM. Source data are provided as a Source Data file.

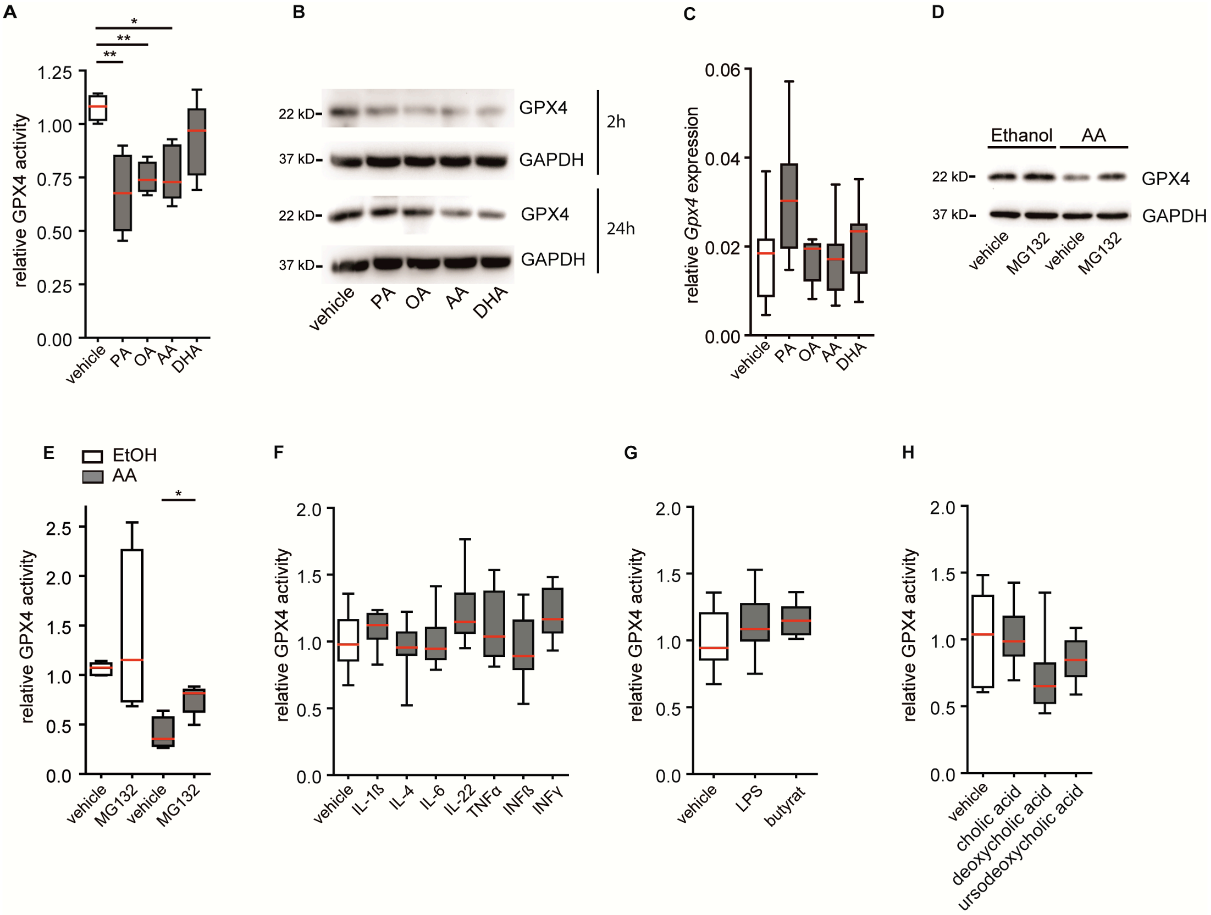

Supplementary Figure 5. Long chain fatty acids impair GPX4 activity in IECs. (A) Relative GPX4 enzymatic activity of MODE-K IECs stimulated with palmitic acid (PA, 100µM), oleic acid (OA, 250µM), arachidonic acid (AA, 20µM), docosahexaenoic acid (DHA, 500µM) or vehicle for 24h. (n=4 biologically independent experiments). *P=0.0112 and **P<0.01. **(B)** Representative GPX4 immunoblot of MODE-K IECs after 2h or 24h stimulation with PA, OA, AA, DHA, or vehicle. GAPDH served as loading control. (n=3 biologically independent experiments). **(C)** Relative *Gpx4* expression of stimulated MODE-K IECs determined by qPCR. (n=8 biologically independent experiments). **(D)** Representative GPX4 western blot of MODE-K IECs after 24h stimulation with AA and the proteasome inhibitor MG132 or vehicle (DMSO). GAPDH served as loading control. (n=3 biologically independent experiments). **(E)** Relative GPX4 enzymatic activity of MODE-K IECs stimulated with arachidonic acid (AA, 20µM) for 24h and with the proteasome inhibitor MG132 or vehicle (DMSO). (n=5 biologically independent experiments). *P=0.0121. Unpaired two-tailed Student's *T*-test. **(F-H)** Relative GPX4 enzymatic activity of MODE-K IECs stimulated for 24h with inflammatory cytokines (IL-1ß 10ng/ml, IL-4 10ng/ml, IL-6 20ng/ml, IL-22 10ng/ml, TNFα 50ng/ml, INFß 2500U/ml and INFγ 50ng/ml), (n=9 biologically independent experiments) **(F)**. lipopolysaccharide (LPS, 100ng/ml) or butyrate (25µM) (n=9 biologically independent experiments) **(G)** or a range of bile acids (1µM) for 24h. (n=4 for vehicle and n=5 for bile acids; biologically independent experiments) **(H)**. For panel **(A), (C)** and **(E-H)** data are presented as boxblot with median and interquartile range (25th and 75th). The whiskers represent minimal and maximal values. One-way ANOVA Bonferroni's multiple comparison test was used for **(A), (C)** and **(F-H)**. Source data are provided as a Source Data file.

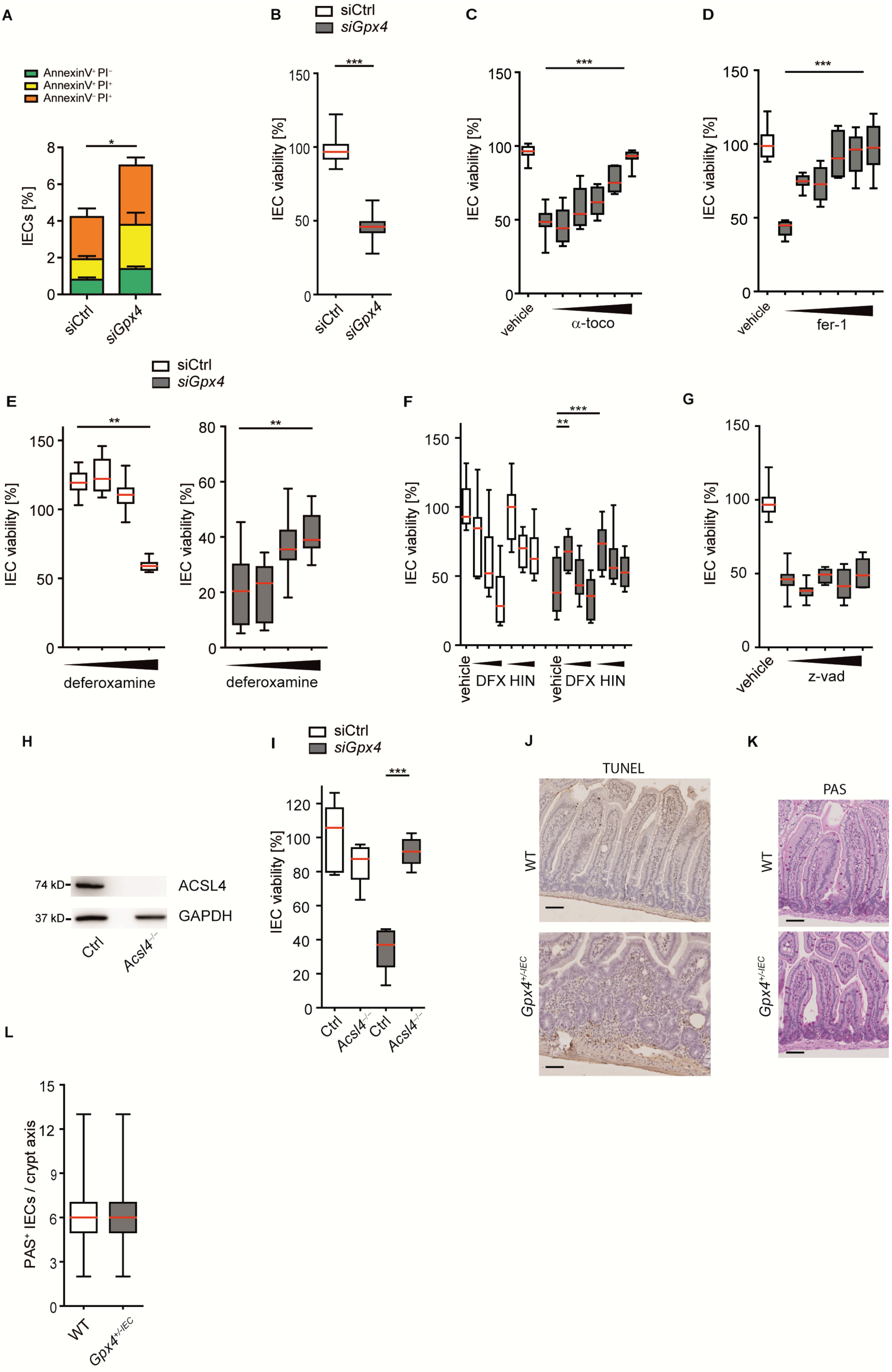
A
AnnexinV+ PI−
AnnexinV+ PI+
AnnexinV− PI+
IECs [%]
siCtrl
siGpx4
B
siCtrl
siGpx4
IEC viability [%]
C
vehicle
α-toco
D
fer-1
E
deferoxamine
F
DFX HIN
G
z-vad
H
74 kD
37 kD
ACSL4
GAPDH
Ctrl
Acsl4−/−
I
J
TUNEL
WT
Gpx4+/-IEC
K
PAS
L
PAS+ IECs / crypt axis

Supplementary Figure 6. IEC death is not a feature of PUFA WD-fed *Gpx4*$^{+/-IEC}$ mice. (A) Quantification of cell death by flow cytometry analysis of Annexin V^+ and propidium iodide$^+$ MODE-K IECs. (n=11 biologically independent experiments). Data presented as mean +/- SEM. *P=0.0177. **(B)** IEC viability assessed by AlamarBlue turn-over. (n=3 biologically independent experiments). ***P<0.001. **(C, D)** Viability quantification of *siGpx4* IECs after stimulation with increasing concentrations of the LPO scavenger α-tocopherol (**C,** 0,01 -1µM) or ferrostatin-1 (**D,** 0,01 -1µM) relative to vehicle-treated siCtrl IECs. (n=3 biologically independent experiments). ***P<0.001. **(E)** Viability quantification of *siGpx4* and siCtrl IECs after stimulation with the iron chelator deferoxamine (DFO, 0,1-20µM). Note that concentrations around 20µM induced death of siCtrl IECs likely due to toxicity. (n=4 biologically independent experiments). **P<0.01. (**F)** Viability quantification of *siGpx4* and siCtrl IECs after stimulation with the iron chelator deferasirox (DFX, 2-10µM) and hinokitol (HIN, 2-10µM). Note that concentrations >2µM induced death of MODE-K IECs likely due to toxicity. (n=2 biologically independent experiments). **P=0.0027 and ***P<0.001. **(G)** Viability quantification of *siGpx4* IECs after stimulation with the pan-caspase inhibitor Z-VAD-FMK (z-vad, 0,1-100µM). Note the lack of protection against cell death in *siGpx4* IECs. (n=3 biologically independent experiments). **(H)** ACSL4 immunoblot of MODE-K cells after CRISPR Cas9-editing of Exon 6 in the *Acsl4* gene. GAPDH served as loading control. (n=1). (**I)** Viability of *siGpx4* IECs with or without deletion of *Acsl4* by CRISPR Cas9 gene editing. Note that *Acsl4* was required for cell death of *siGpx4* IECs. (n=3 biologically independent experiments). ***P<0.001. **(J)** Representative images of TUNEL-labelled sections of indicated genotypes after a 3-month PUFA WD. Note that lamina propria immune cells, but no IECs, are TUNEL-labelled (brown). (n=5 mice per group). Scale bars indicate 50µm. **(K, L)** Representative images of periodic acid-Schiff (PAS) reaction in *Gpx4*$^{+/-IEC}$ and WT mice fed a PUFA WD **(K)**, with quantification of PAS^+ goblet cells. **(L)** Reduced goblet cell numbers were neither observed in the inflamed nor uninflamed mucosa of *Gpx4*$^{+/-IEC}$ mice. (n=4 mice per group). Scale bars indicate 50µm. For panel **(B-G)**, **(I)** and **(L)** data are presented as boxblot with median and interquartile range (25th and 75th). The whiskers represent minimal and maximal values. Unpaired two-tailed Student's *T*-test was used for **(A)**, **(B)** and **(L)**. For panel **(C-G)** and **(I)** an one-way ANOVA with Bonferroni's multiple comparison test was used. Source data are provided as a Source Data file.

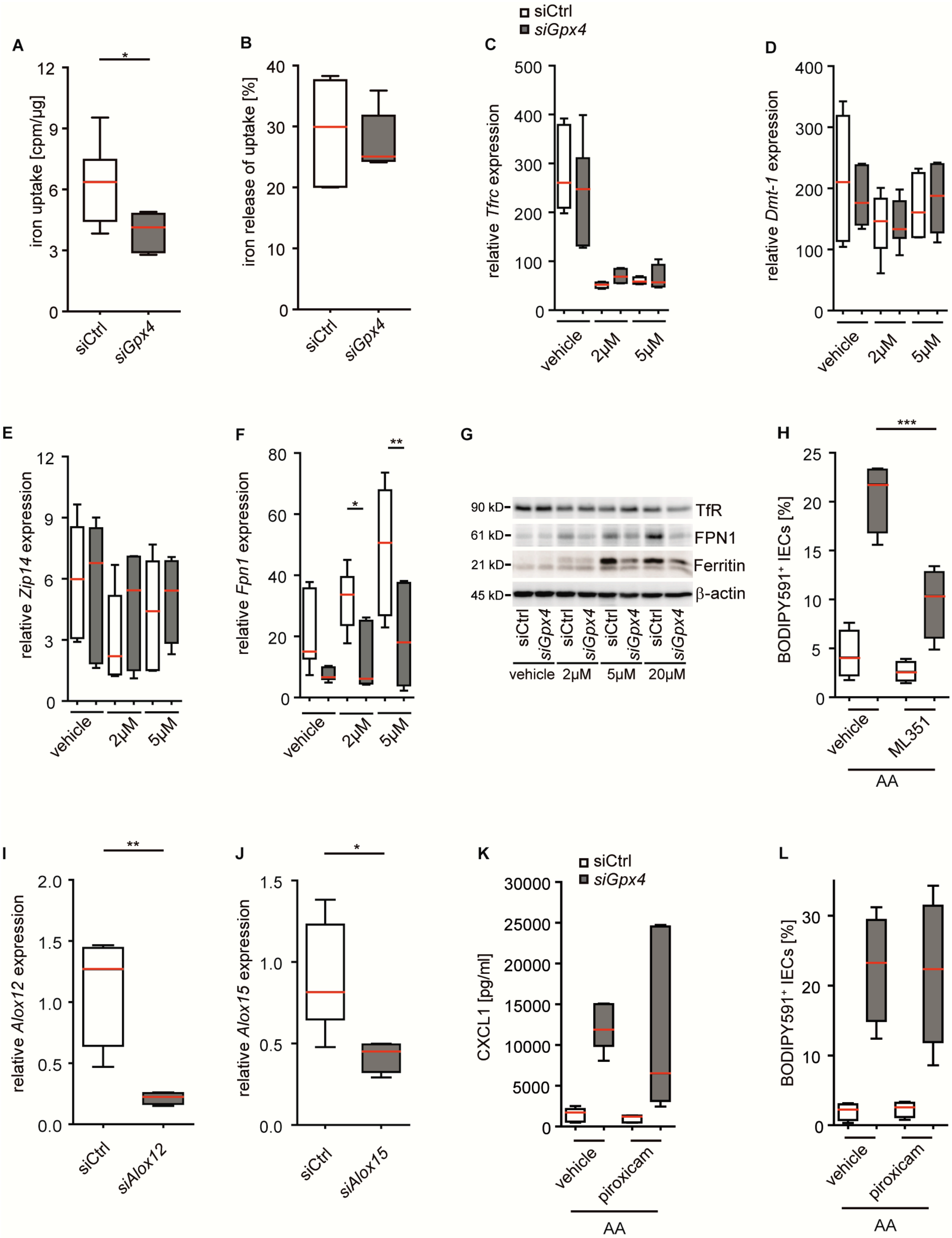

Supplementary Figure 7. GPX4 is required for uptake of ferric iron that cannot be explained by differential regulation of iron transporters. (A) Iron uptake of radioactively labelled iron(III) in *siGpx4* and siCtrl IECs after 2 hours. (n=3 biologically independent experiments). *P=0.0269. **(B)** ^{59}Fe release relative to uptake in *siGpx4* and siCtrl IECs. (n=3 biologically independent experiments). **(C-F)** Relative expression of *Tfrc*, *Dmt-1, Zip14* and *Fpn1* in *siGpx4* and siCtrl IECs after stimulation with Fe(III) sulphate (2µM or 5µM) or vehicle for 6h. (n=6 for vehicle and n=4 for Fe stimulations, biologically independent experiments).

*P=0.0483 and **P=0.0018 for **(F)**. **(G)** Representative immunoblot of the transferrin receptor (TfR), ferroportin (FPN1) and ferritin in *siGpx4* and siCtrl MODE-K IECs after 6h stimulation with Fe(III) sulphate (2 µM, 5 µM, 20µM) or vehicle. ß-Actin served as loading control. (n=3 biologically independent experiments). **(H)** LPO quantification by flow cytometry of BODIPY581 C11^{+} si*Gpx4* or siCtrl IECs stimulated with AA (20µM) and co-treated with the LOX-12/15 inhibitor ML351 (10µM) as compared to vehicle (DMSO) for 24h. (n=4 biologically independent experiments). ***P<0.001. **(I)** Relative *Alox12* expression of siRNA silenced MODE-K IECs determined by qPCR. (n=4 biologically independent experiments). **P=0.0073. **(J)** Relative *Alox15* expression of siRNA silenced MODE-K IECs determined by qPCR. (n=5 for siCtrl and n=4 for *siAlox15,* biologically independent experiments). *P=0.0268. **(K)** Quantification of CXCL1 in the supernatant from *siGpx4* and siCtrl IECs stimulated with AA (20µM) and co-treated with the COX1/2 inhibitor piroxicam (20µM) for 24h. (n=7 for siCtrl and n=5 for *siGpx4,* biologically independent experiments). **(L)** LPO quantification by flow cytometry of BODIPY581/591 C11^{+} *siGpx4* or siCtrl IECs stimulated with AA (20µM) and co-treated with the COX1/2 inhibitor piroxicam (20µM) for 24h. (n=4 biologically independent experiments). For panel **(A-F)** and **(H-L)** data are presented as boxblot with median and interquartile range (25th and 75th). The whiskers represent minimal and maximal values. Unpaired two-tailed Student's *T*-test was used for **(A)**, **(B)**, **(I)** and **(J)**. For panel **(C-F)**, **(H)**, **(K)** and **(L)** an one-way ANOVA with Bonferroni's multiple comparison test was used. Source data are provided as a Source Data file.

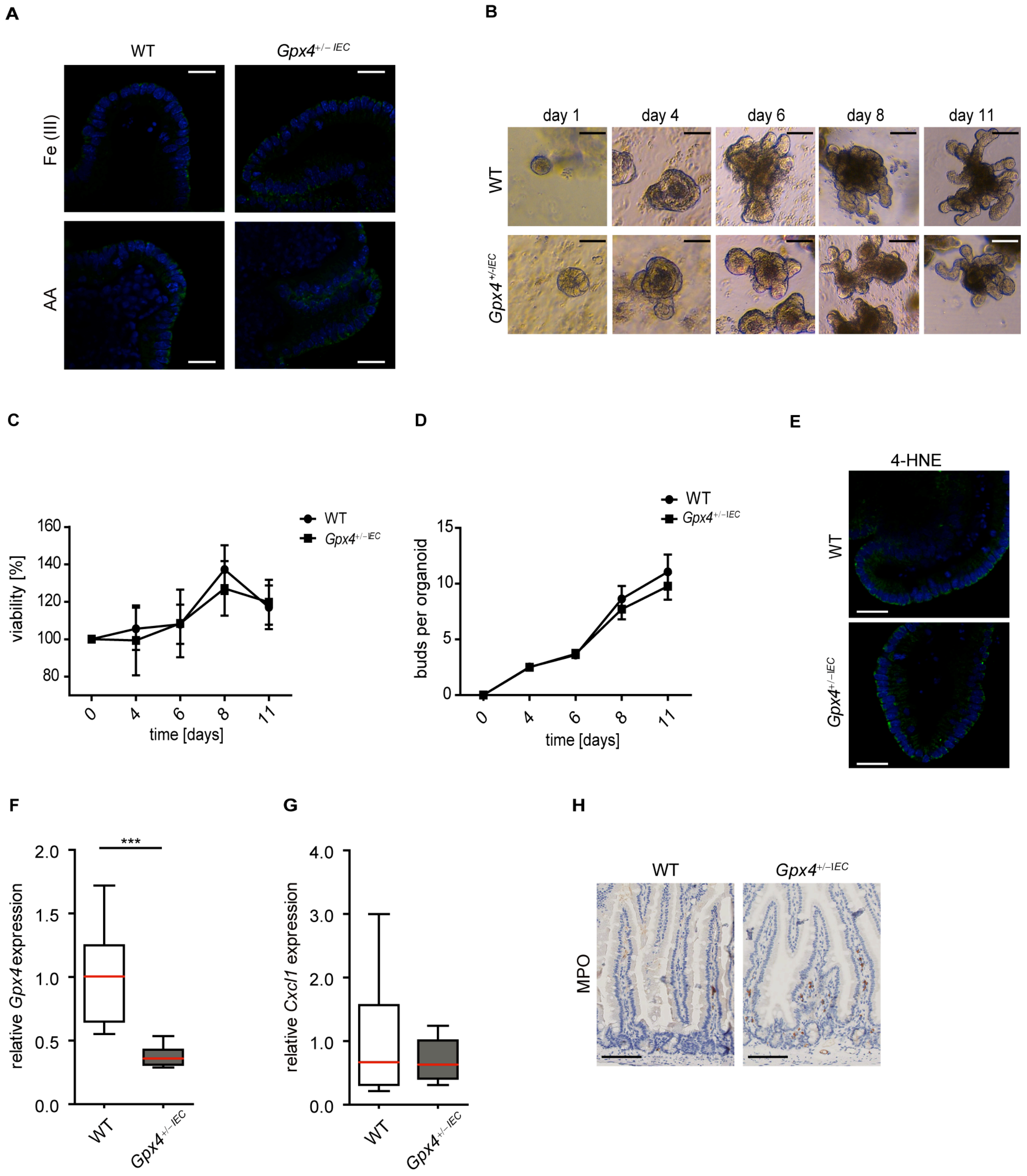

Supplementary Figure 8. AA and FM exposure triggers MPO^{+} cell infiltration in *$Gpx4^{+/-IEC}$* mice. (A) Representative confocal microscopy images of 4-HNE labelled organoids (green) from indicated genotypes after stimulation with AA or ferric iron for 24h. Scale bars indicate 20µm. (n=3 biologically independent samples). **(B)** Representative light microscopy images of organoids from WT and *$Gpx4^{+/-IEC}$* mice over the course of 11 days (n=3 biologically independent samples). Scale bars indicates 100µm. **(C, D)** Quantification of viable organoids assessed by morphological means **(C)** and buds per organoid **(D)**. (n=3 biologically independent samples). Data presented as mean +/- SEM. **(E)** Representative immunofluorescence images of 4-HNE labelled (green) organoids from indicated genotypes. Scale bars indicate 50µm. (n=3 biologically independent samples). **(F)** Relative *Gpx4* expression in WT and *$Gpx4^{+/-IEC}$* organoids determined by qPCR. (n=8 biologically independent samples). ***P<0.001. **(G)** Relative *Cxcl1* expression in WT and *$Gpx4^{+/-IEC}$* organoids. (n=6 biologically independent samples). **(H)** Representative immunohistochemistry images of

MPO^+ cells (brown) in indicated genotypes orally exposed to AA and FM. (n=6 biologically independent samples). Scale bar indicates 100µm. For panel **(F)** and **(G)** data are presented as boxblot with median and interquartile range (25th and 75th). The whiskers represent minimal and maximal values. Unpaired two-tailed Student's *T* –test. Source data are provided as a Source Data file.

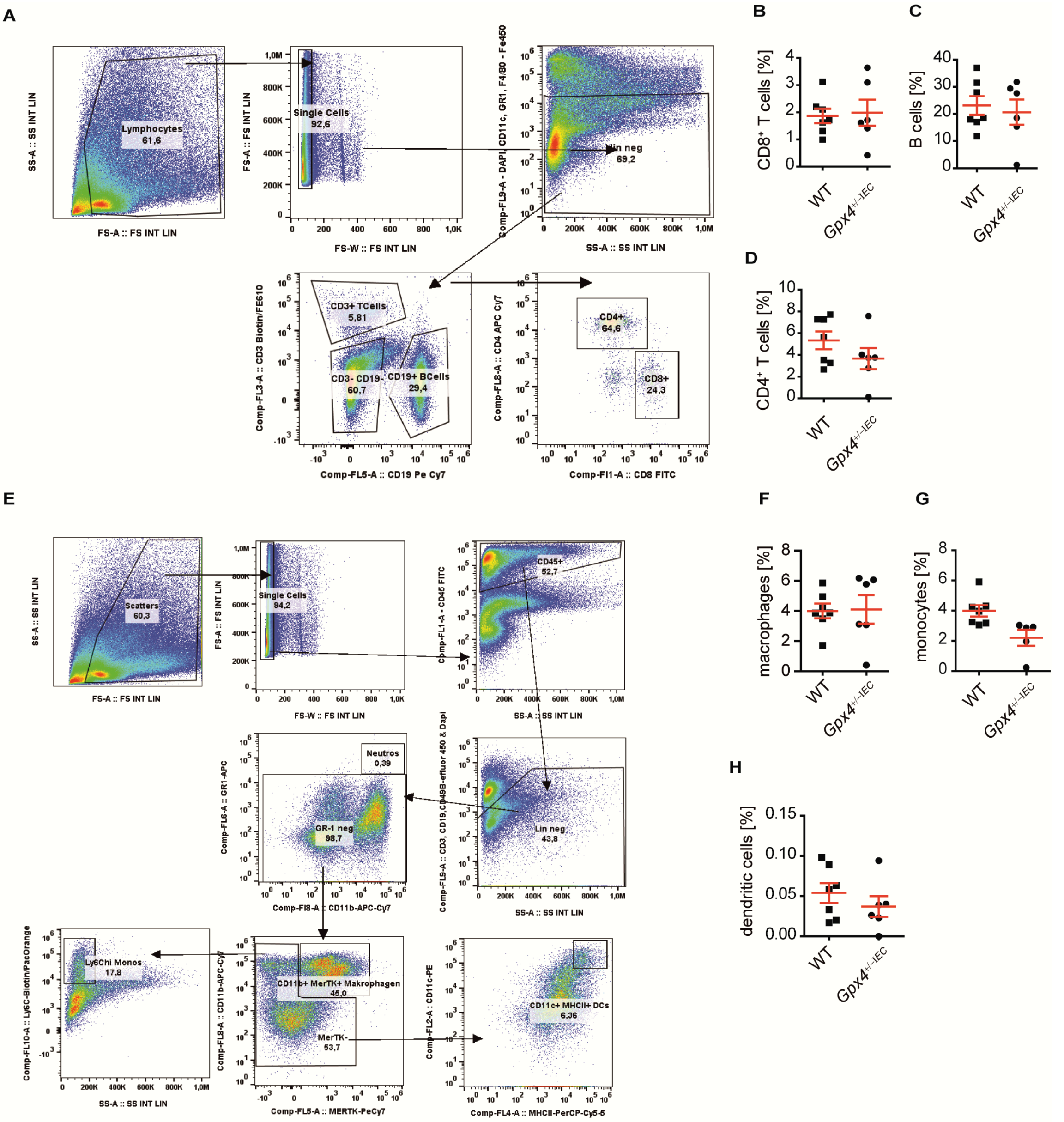

Supplementary Figure 9. Quantification of innate and adaptive immune cells by flow cytometry analysis after AA/FM exposure of *Gpx4*$^{+/-IEC}$ and WT mice. (A) Gating strategy for lamina propria infiltrating adaptive immune cells. **(B-D)** Percentage of indicated adaptive immune cells in relation to lineage negative cells. (n=7 mice for WT and n=6 mice for *Gpx4*$^{+/-IEC}$). Data presented as mean +/- SEM. **(E)** Gating strategy for lamina propria infiltrating innate immune cells. **(F-H)** Percentage of indicated innate immune cells in relation to all CD45^{+} cells. Each dot represents an experimental animal (n=7 mice for WT and n=6 mice for *Gpx4*$^{+/-IEC}$). Data presented as mean +/-SEM. Unpaired two-tailed Student's *T*-test was used for **(B-D)** and **(F-H).** Source data are provided as a Source Data file.

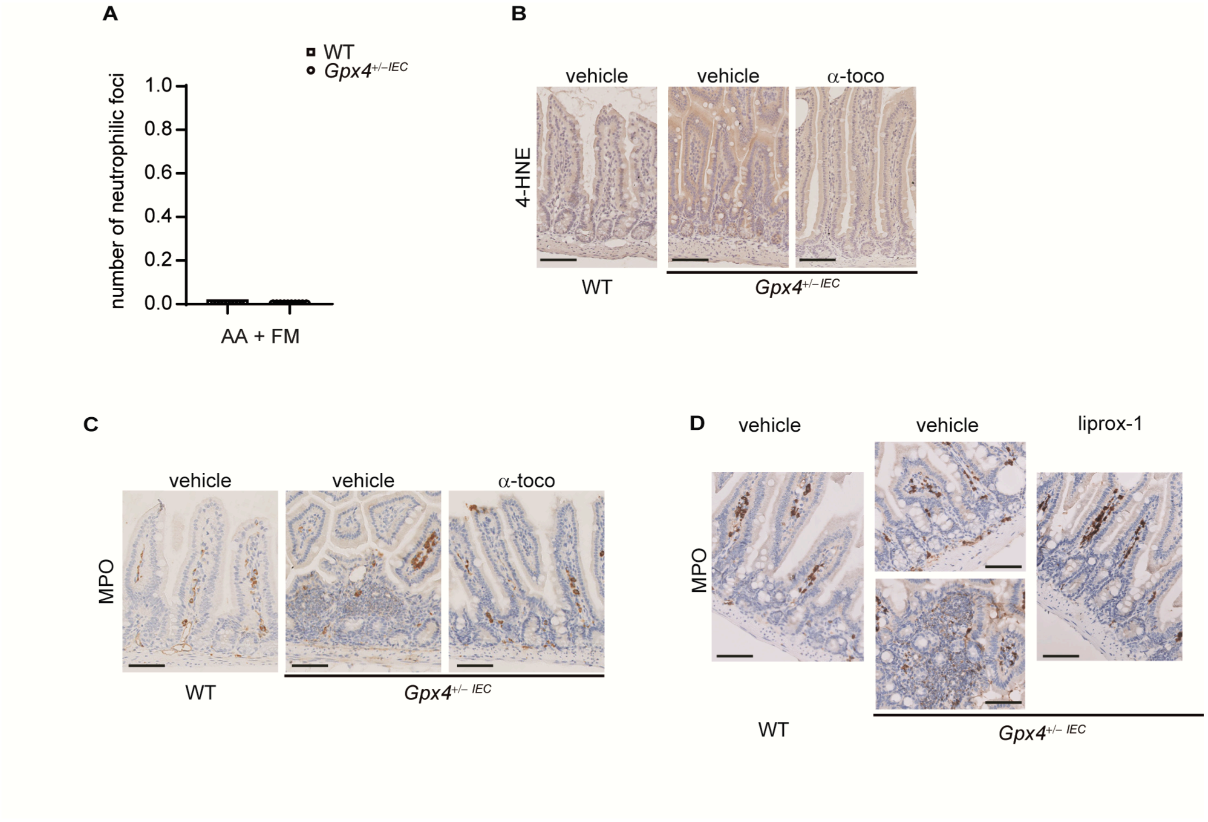

Supplementary Figure 10. α-tocopherol and liproxstatin-1 protect against PUFA WD-induced enteritis in *Gpx4*$^{+/-IEC}$ mice. (A) Histology score in the colon of indicated genotypes orally exposed to ferric maltol and AA. Each dot represents one experimental animal. (n=10 mice per group). **(B, C)** Representative images of 4-HNE immunoreactivity (brown), indicative for LPO **(B)**, and MPO^{+} cells **(C)** in *Gpx4*$^{+/-IEC}$ and WT mice on a PUFA-enriched WD with or without supplementation of α-tocopherol in the drinking water. (n=5 mice per group). Scale bars indicate 100µm. **(D)** Representative images of MPO^{+} cells (brown) in *Gpx4*$^{+/-IEC}$ and WT mice on a PUFA-enriched WD with or without liproxstatin-1 treatment. (n=5 mice per group). Scale bars indicate 100µm. Data presented as mean +/-SEM. Unpaired two-tailed Student's *T*-test was used for **(A)**. Source data are provided as a Source Data file.

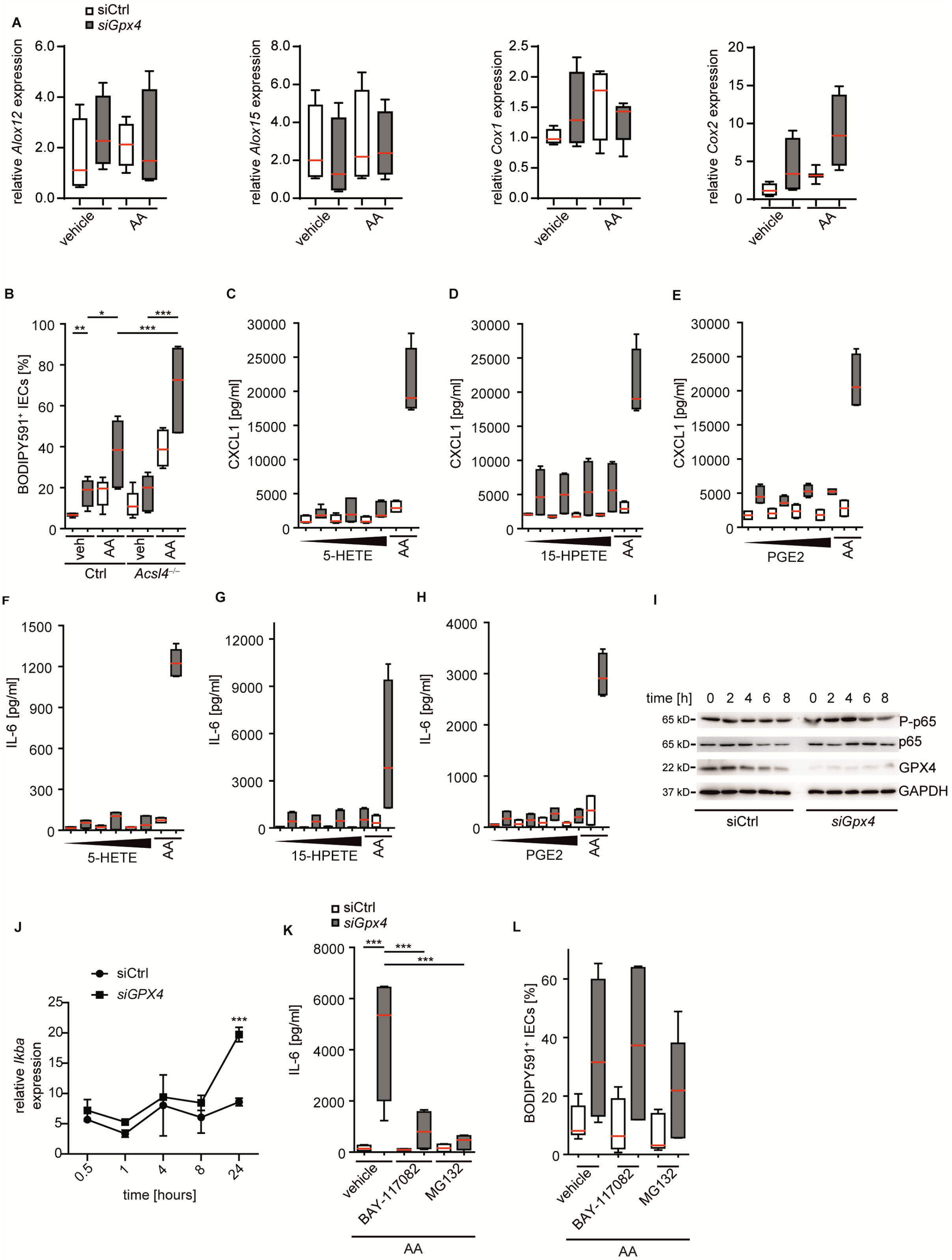

Supplementary Figure 11. AA evokes NF-κB activation and NF-κB inhibition ameliorates AA-induced cytokine responses. (A) Relative expression of *Alox12*, *Alox15*, *Cox1* and *Cox2* from *siGpx4* and siCtrl IECs stimulated with AA or vehicle. (n=4 for *Alox12*, *Alox15* and *Cox2* and n=5 for *Cox1;* biologically independent experiments). **(B)** LPO quantification by flow cytometry of BODIPY581 C11^{+} si*Gpx4* or siCtrl IECs with or without deletion of *Acsl4* and stimulated with AA (20µM) for 24h. (n=6 biologically independent experiments). **P<0.01,

***P<0.001. **(C-H)** Quantification of CXCL1 **(C-E)** and IL-6 **(F-H)** in the supernatant from *siGpx4* and siCtrl IECs stimulated with increasing concentrations of abundant LOX (5-HETE, 15-HPETE) and COX (PGE2) metabolites for 24h. AA stimulation served as positive control. Note that neither LOX nor COX metabolites induce a cytokine response in *siGpx4* or siCtrl IECs. (n=4 biologically independent experiments). **(I)** Representative immunoblot of phospho-NF-κB p65 in *siGpx4* and siCtrl MODE-K IECs after stimulation with AA (20μM) and Fe(III) sulphate (5μM) over a time-course of 8h. GAPDH served as loading control. (n=3 biologically independent experiments). **(J)** Quantification of *Iκba* expression, a transcript regulated by NF-κB, from *siGpx4* and siCtrl IECs over a course of AA or vehicle stimulation determined by qPCR. (n=3 biologically independent experiments). Data presented as mean +/- SEM. Two-way ANOVA with Bonferroni's multiple comparison test. ***P<0.001. **(K)** Quantification of IL-6 in the supernatant from *siGpx4* and siCtrl IECs stimulated with AA (20μM) and the NF-κB inhibitors BAY-117082 (10μM) and MG132 (125nM) or vehicle for 24h. (n=3 biologically independent experiments). ***P<0.001. **(L)** LPO quantification in *siGpx4* or siCtrl IECs stimulated with AA (20μM) and the NF-κB inhibitors BAY-117082 (10μM) and MG132 (125nM) or vehicle for 24h. (n=4 biologically independent experiments). For panel **(A-H), (K)** and **(L)** data are presented as boxblot with median and interquartile range (25th and 75th). The whiskers represent minimal and maximal values. One-way ANOVA with Bonferroni's multiple comparison test was used. Source data are provided as a Source Data file.

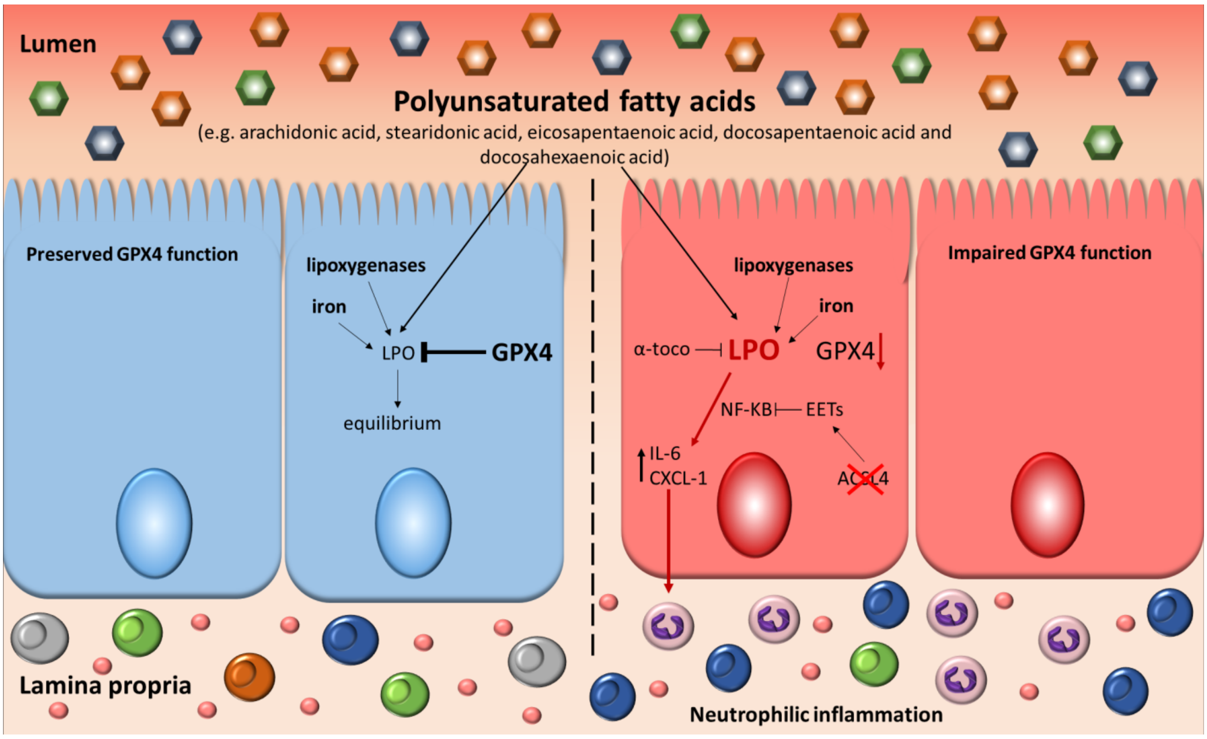

Supplementary Figure 12. Model of PUFA-induced and GPX4-restricted enteritis. Left panel: Exposure of polyunsaturated fatty acids (PUFAs) such as arachidonic acid (AA) induces LOX-mediated lipid peroxidation (LPO) that is fuelled by iron availability. GPX4 critically restricts epithelial LPO to maintain gut homeostasis [1]. **Right panel:** Reduced intestinal epithelial GPX4 activity is a feature of ileal Crohn's disease and may be evoked by yet unidentified inflammatory cues. In IECs with reduced GPX4 activity, PUFAs such as AA promote lipoxygenase-driven LPO and cytokine production (i.e. CXCL1 and IL-6 expression), which are fuelled by iron availability and ameliorated by the LPO scavenger α-tocopherol. ACSL4 may limit a PUFA-induced cytokine response in IECs with reduced GPX4 activity by modulation of AA metabolism. As such, similar mechanisms that control ferroptosis [1] also control epithelial cytokine responses of IECs with reduced GPX4 activity. A PUFA Western diet triggers granuloma-like neutrophilic intestinal inflammation resembling aspects of human CD which can be ameliorated by α-tocopherol treatment. As such, GPX4-restricted LPO emerges as a rheostat of PUFA-induced neutrophilic inflammation in the intestine which is fuelled by dietary lipids.

Supplementary Tables

Supplementary Table 1: Expected (Mendelian ratios) and observed genotypes from *Gpx4 Villin Cre* breeding. Expected (Mendelian ratios) and observed genotypes after cross breeding *Gpx4*$^{fl/wt}$ *Villin Cre*$^{+}$ and *Gpx4*$^{fl/fl}$ *Villin Cre*$^{-}$ parents fed with an α-tocopherol -enriched diet. Overall, 74 new-born animals were analysed.

Gpx4$^{fl/wt}$ *Villin Cre*$^{+}$<br>X<br>*Gpx4*$^{fl/fl}$ *Villin Cre*$^{-}$	*Gpx4*$^{fl/wt}$<br>*Villin Cre*$^{-/}$	*Gpx4*$^{fl/wt}$<br>*Villin Cre*$^{+}$	*Gpx4*$^{fl/fl}$<br>*Villin Cre*$^{-}$	*Gpx4*$^{fl/fl}$<br>*Villin Cre*$^{+}$
Expected	25%	25%	25%	25%
Observed	30%	32%	27%	11%

Supplementary Table 2. Relative polyunsaturated fatty acid abundance in the Western diet (WD) and the WD supplemented with 10% fish oil (PUFA WD).

Fatty acid	WD [%]	PUFA WD [%]
C18:2 (n6)	0,38	0,41
C18:3 (n3)	0,11	0,15
C18:4 (n3)	-	0,30
C20:4 (n6)	-	0,09
C20:5 (n3)	-	1,75
C22:5 (n3)	-	0,19
C22:6 (n3)	-	1,21

Supplementary Table 3. Lipid mediators [pg/million cells] determined by LC-MS/MS. Samples were obtained from siCtrl and *siGpx4* MODE-K IECs with or without co-deletion of *Acsl4*$^{-/-}$ and analyzed following stimulation with AA or vehicle for 24h. Three independent experiments were performed and pooled for the analysis.

			COX		15-LOX/5-LOX		5-LOX		15-LOX		12-LOX
			TXB2	6-K-PGF1A	PGE2	LXB4	LXA4	LTB4	5-HETE	15-HETE	12-HETE
WT	vehicle	siCtrl	42,04	16,15	1081,85	39,19	0,70	-	21,83	326,15	39,33
		siGpx4	59,27	17,77	1956,48	39,32	0,00	0,13	21,96	569,22	39,40
	AA	siCtrl	187,07	105,14	8283,86	42,15	5,51	-	583,06	3663,83	568,19
		siGpx4	241,29	129,45	9992,21	13,13	2,05	-	455,87	4338,66	472,38
Acsl4$^{-/-}$	vehicle	siCtrl	112,76	84,33	4519,04	64,05	0,54	0,26	47,10	1039,80	57,85
		siGpx4	123,74	103,39	5955,85	49,22	0,87	-	67,26	1527,85	80,87
	AA	siCtrl	498,36	426,47	27475,92	135,27	7,44	0,76	3670,79	9504,63	1072,46
		siGpx4	431,44	398,61	27953,24	118,42	11,01	-	2645,51	8468,00	686,80

Supplementary Table 3 continued.

			CYP450							
			14,15-DHET	11,12-DHET	8,9-DHET	5,6-DHET	14,15-EET	11,12-EET	8,9-EET	5,6-EET
WT	vehicle	siCtrl	1,11	4,03	-	-	11,05	12,82	99,73	84,57
		siGpx4	1,50	5,01	1,57	-	38,88	10,54	145,25	79,39
	AA	siCtrl	1404,18	878,23	400,89	24,89	1801,47	445,98	671,40	394,19
		siGpx4	812,48	754,14	240,99	16,21	1850,24	558,16	518,50	218,72
Acsl4$^{-/-}$	vehicle	siCtrl	2,06	3,51	-	-	24,60	-	228,78	233,70
		siGpx4	2,12	10,07	-	-	9,08	12,15	251,82	94,74
	AA	siCtrl	2478,41	3133,22	1374,39	96,98	6648,23	2349,70	1977,08	1655,59
		siGpx4	1829,65	2542,62	913,63	70,73	5630,30	1830,04	1838,77	1401,31

Supplementary Table 4. Sequences of qPCR primer pairs and antibodies for flow cytometry.

Mouse	Forward	Reverse
ß-act	GATGCTCCCCGGGCTGTATT	GGGGTACTTCAGGGTCAGGA
gpx4	TGTGCATCCCGCGATGATT	CCCTGTACTTATCCAGGCAGA
il-6	AAGTGCATCATCGTTGTTCATACA	TGTTCTCTGGGAAATCGTGGA
cxcl-1	CTGGGATTCACCTCAAGAACATC	CAGGGTCAAGGCAAGCCTC
fpn-1	ACCAAGGCAAGAGATCAAACC	AGACACTGCAAAGTGCCACAT
dmt-1	TACCTAGACCCAGGAAACATCG	CACTCCAAGTCTCGCTGCAA
tfrc-2	TTGGGGTCTACTTCGGAGAGT	GACAGGAGCCTAAGTGCTCAG
zip14	CACATTAGCCTTGGCCTC	GAGATCGCTCGCTCAAGT
iκba	TGAAGGACGAGGAGTACGAGC	TTCGTGGATGATTGCCAAGTG
alox12	GTTCCACACATCCGTTACACT	CCGAGTAAGCAACTGAACATGG
alox15	GGCTCCAACAACGAGGTCTAC	CCCAAGGTATTCTGACACATCC
cox1	ATGAGTCGAAGGAGTCTCTCG	GCACGGATAGTAACAACAGGGA
cox2	TGCACTATGGTTACAAAAGCTGG	TCAGGAAGCTCCTTATTTCCCTT

Human	Forward	Reverse
GPX4	GAGGCAAGACCGAAGTAAACTAC	CCGAACTGGTTACACGGGAA
GAPDH	GTCGCCAGCCGAGCC	CCCAATACGACCAAATCCGT

Antibody/Fluorophore	Source	Catalog number	Clone	Dilution
MERTK-PE/CY7	eBioscience	Cat# 25-5751-82	DS5MMER	400
CD11b-APC/eFluor780	eBioscience	Cat# 47-0112-82	M1/70	400
CD11c-PE	Biolegend	Cat# 117308	N418	400
CD45-FITC	eBioscience	Cat# 11-0454-82	104	400
LY6c-Biotin	Biolegend	Cat# 128003	HK1.4	400
Streptavidin Pacific Orange	Invitrogen	S32365	N/A	800
GR1-APC	Biolegend	Cat# 108412	RB6-8C5	400
MHCII-PerCP Cy5-5	Biolegend	Cat# 116416	AF6-120.1	400
DAPI	Biolegend	422801	N/A	40000
CD3-Biotin	eBioscience	Cat# 13-0032-82	17A2	400
Streptavidin FE610	eBioscience	61-4317-82	N/A	800
CD4-APC/eFluor 780	eBioscience	Cat# 47-0041-82	GK1.5	400
CD19-PE/Cy7	Biolegend	Cat# 115520	6D5	400
CD8-FITC	BD Bioscience	Cat# 553030	53-6.7	400
CD45-APC	Biolegend	Cat# 103112	30-F11	400
CD3-eFluor450	eBioscience	17A2 48-0032-8	17A2	400
CD19-eFluor450	eBioscience	Cat# 48-0193-82	eBio1D3	400
CD49b-eFluor450	eBioscience	Cat# 48-5971-82	DX5	400
GR1+-eFluor450	eBioscience	Cat# 48-5931-82	RB6	400

CD11c-eFluor450	eBioscience	Cat# 48-0114-82	N418	400
F4/80-eFluor450	eBioscience	Cat# 48-4801-82	BM8	400
AnnexinV-FITC	BD Biosciences	Cat# 556419	N/A	400
7-AAD	BD Pharmingen	51-68981E	N/A	400
Propidium iodide	BD Pharmingen	51-66211E	N/A	1000

References

1 Stockwell, B. R. *et al.* Ferroptosis: A Regulated Cell Death Nexus Linking Metabolism, Redox Biology, and Disease. *Cell* **171**, 273-285, doi:10.1016/j.cell.2017.09.021 (2017).
