# Supplementary material for: Dietary lipids fuel GPX4-restricted enteritis resembling Crohn’s disease
Source: Nat Commun. 2020 Apr 14;11:1775. doi: 10.1038/s41467-020-15646-6 (PMC7156516; doi:10.1038/s41467-020-15646-6)
Supplement: Supplementary file 3 — Reporting Summary [file 41467_2020_15646_MOESM3_ESM.pdf]

nature research

Corresponding author(s): Timon-Erik Adolph

Last updated by author(s): Mar 9, 2020

Reporting Summary

Nature Research wishes to improve the reproducibility of the work that we publish. This form provides structure for consistency and transparency in reporting. For further information on Nature Research policies, see Authors & Referees and the Editorial Policy Checklist .

Statistics

For all statistical analyses, confirm that the following items are present in the figure legend, table legend, main text, or Methods section.

n/a	Confirmed	
☐	☒	The exact sample size (*n*) for each experimental group/condition, given as a discrete number and unit of measurement
☐	☒	A statement on whether measurements were taken from distinct samples or whether the same sample was measured repeatedly
☐	☒	The statistical test(s) used AND whether they are one- or two-sided<br>*Only common tests should be described solely by name; describe more complex techniques in the Methods section.*
☒	☐	A description of all covariates tested
☐	☒	A description of any assumptions or corrections, such as tests of normality and adjustment for multiple comparisons
☐	☒	A full description of the statistical parameters including central tendency (e.g. means) or other basic estimates (e.g. regression coefficient) AND variation (e.g. standard deviation) or associated estimates of uncertainty (e.g. confidence intervals)
☐	☒	For null hypothesis testing, the test statistic (e.g. *F*, *t*, *r*) with confidence intervals, effect sizes, degrees of freedom and *P* value noted<br>*Give P values as exact values whenever suitable.*
☒	☐	For Bayesian analysis, information on the choice of priors and Markov chain Monte Carlo settings
☒	☐	For hierarchical and complex designs, identification of the appropriate level for tests and full reporting of outcomes
☒	☐	Estimates of effect sizes (e.g. Cohen's *d*, Pearson's *r*), indicating how they were calculated

Our web collection on statistics for biologists contains articles on many of the points above.

Software and code

Policy information about availability of computer code

Data collection	Zen Black, Gallios Software
Data analysis	Zen 2012 software, FlowJo V10, MassHunter Quantitative Analysis Software, GraphPad Prism v 5.04

For manuscripts utilizing custom algorithms or software that are central to the research but not yet described in published literature, software must be made available to editors/reviewers. We strongly encourage code deposition in a community repository (e.g. GitHub). See the Nature Research guidelines for submitting code & software for further information.

Data

Policy information about availability of data

All manuscripts must include a data availability statement. This statement should provide the following information, where applicable:

- Accession codes, unique identifiers, or web links for publicly available datasets
- A list of figures that have associated raw data
- A description of any restrictions on data availability

The data of this study are available from the corresponding author upon reasonable request. The dataset generated in this study are deposited in a publicly available platform (https://data.mendeley.com, DOI: 10.17632/k9ync2kd3g.2; Data for: Dietary lipids fuel GPX4-restricted enteritis resembling Crohn’s disease). The source data for Figure 1-6 and Supplementary Figure S1-11 are provided in the Source Data File.

Field-specific reporting

Please select the one below that is the best fit for your research. If you are not sure, read the appropriate sections before making your selection.

Life sciences study design

All studies must disclose on these points even when the disclosure is negative.

Sample size	Sample-size was choosen according to published experiments and our own experience (PMID: 24089213). At least three independent replicates were done for experiments, except otherwise stated. Power-analysis was performed for in-vivo experiments.
Data exclusions	Data were only exluded when experiments failed due to technical errors. Patients were excluded when histology report conflicted with endoscopic report.
Replication	At least three independet replicates were done for experiments, as indicated in figure legends. Reproduction was reliable in all experiments.
Randomization	Healthy controls and IBD patients were defined by endoscopic and histological assessment. Animals were allocated randomly into the experimental groups, machted to age, sex and genotype.
Blinding	Histopathological analysis (Scoring, evaluation of IHC/IF) and LC-MS/MS analysis was performed in a blinded fashion.

Reporting for specific materials, systems and methods

We require information from authors about some types of materials, experimental systems and methods used in many studies. Here, indicate whether each material, system or method listed is relevant to your study. If you are not sure if a list item applies to your research, read the appropriate section before selecting a response.

Materials & experimental systems

n/a	Involved in the study	
☐	☒	Antibodies
☐	☒	Eukaryotic cell lines
☒	☐	Palaeontology
☐	☒	Animals and other organisms
☐	☒	Human research participants
☒	☐	Clinical data

Methods

n/a	Involved in the study	
☒	☐	ChIP-seq
☐	☒	Flow cytometry
☒	☐	MRI-based neuroimaging

Antibodies

Antibodies used	anti-GPX4 Abcam ab125066 1:400 (IHC), 1:2000 (WB); anti-ACSL4 Abcam ab155282 1:1000; anti-4HNE Abcam ab46545 1:200 (IHC), 1:2000 (WB); anti-GR1 Biolegend 108413 1:100; anti-rabbit IgG Alexa Fluor 488 Invitrogen, A-11034 1:1000; anti-rat IgG Alexa Fluor 594 Invitrogen, A-11007 1:1000; anti-MPO Dako, IS511 1:200; anti-TfR Invitrogen H68.4 1:1000; anti-FPN1 Eurogentec 1:1000; anti-ferritin Sigma F5012 1:1000; anti-phospho-NFKB p65 Cell Signalling Technology #3039 1:1000; anti-NFKB p65 Cell Signaling Technology #8242 1:1000; anti-GAPDH Cell Signalling Technology 14C10 1:2000; anti-ß-actin Sigma A2066 1:1000; MERTK-PE/CY7 eBioscience DS5MMER 25-5751-82 1:400; CD11b-APC/eFluor780 eBioscience M1/70, 47-0112-82 1:400; CD11c-PE Biolegend N418, 117308 1:400; CD45-FITC eBioscience 104, 11-0454-82 1:400; LY6c-Biotin Biolegend HK1.4 128003 1:400; Streptavidin Pacific Orange Invitrogen S32365 1:800; GR1-APC Biolegend RB6-8C5, 108412 1:400; MHCII-PerCP Cy5-5 Biolegend AF6-120.1, 116416 1:400; DAPI Biolegend 422801 1:40000; CD3-Biotin eBioscience 17A2 13-0032-82 1:400; Streptavidin FE610 eBioscience 61-4317-82 1:800; CD4-APC/eFluor 780 eBioscience GK1.5, 47-0041-82 1:400; CD19-PE/Cy7 Biolegend 6D5, 115520 1:400; CD8-FITC BD Bioscience 53-6.7, 553030 1:400; CD45-APC Biolegend 30-F11 103112 1:400; CD3-eFluor450 eBioscience 17A2 48-0032-8 1:400; CD19-eFluor450 eBioscience eBio1D3 48-0193-82 1:400; CD49b-eFluor450 eBioscience DX5 48-5971-82 1:400; GR1+-eFluor450 eBioscience RB6-8C5 48-5931-82 1:400; CD11c-eFluor450 eBioscience N418 48-0114-82 1:400; F4/80-eFluor450 eBioscience BM8 48-4801-82 1:400; AnnexinV-FITC BD Biosciences 556419 1:400; 7-AAD BD Pharmingen 51-68981E 1:400; Propidium iodide BD Pharmingen 51-66211E 1:1000; secondary biotinylated antibody for IHC Vector MP-7401 Ready to use; HRP-conjugated secondary antibody Cell Signalling Technology 7074 1:2000
Validation	For validation of antibodies and commercially available assays we refer to datasheets of the manufacturer. GPX4 antibody was validated by the lack of staining of GPX4 KO cells.

Eukaryotic cell lines

Policy information about cell lines

Cell line source(s)	MODE-K Cell line was provided by D. Kaiserlian; HEK293 were purchased from ATCC (CRL-1573).
Authentication	HEK293 cells were purchased from ATCC and cultured according to recommandations, MODE-K Cell line was provided by D. Kaiserlian. MODE-K cells were authenticated by electron microscopy and light microscopy appearance, similar to HEK293 cells.

Mycoplasma contamination	Cells were tested negative for mycoplasma.
Commonly misidentified lines (See ICLAC register)	No cell lines used are listed in the ICLAC database.

Animals and other organisms

Policy information about studies involving animals; ARRIVE guidelines recommended for reporting animal research

Laboratory animals	Male and female mice were used, all included mice had a C57BL6/J background. GPX4flox/flox mice were crossed with VillinCre positive mice to obtain GPX4flox/wt VillinCre+ and GPX4flox/wt VillinCre- mice. Mice were 7 to 10 week old at the start of the experiments. Housing was done under SPF conditions with a 12/12 hour dark/light cycle.
Wild animals	The study does not involve wild animals.
Field-collected samples	The study does not involve samples collected from the field.
Ethics oversight	Animal experiments were performed in accordance with institutional guidelines of the Medical University of Innsbruck and following approval by federal authorities.

Note that full information on the approval of the study protocol must also be provided in the manuscript.

Human research participants

Policy information about studies involving human research participants

Population characteristics	Healthy Controls: N: 21 (female 57%), Age: 51,62 (+-12,1), BMI: 28,56 (+- 6,17),<br>CD: N: 16 (female 50%), Age: 39,06 (+- 14,4), BMI 24,06(+-5,81), Harvey Bradshaw: 4,67 (+-0,52), CRP: 0,55 [mg/dl] (+-0,04)<br>UC: N: 8 (female 25%), Age: 40.38 (+-13,7), BMI: 23,7 (3,48), Mayo: 4,75 (+- 0,69), CRP: 0,59 [mg/dl] (+-0.14)
Recruitment	Patients and healthy controls were recruited in the outpatient clinic and informed consent was obtained as stated in the method section. Inclusion criteria were very broad (see methods), and endoscopic staff was neither involved in experimentation or analysis of the samples nor informed about the research question, such that selection bias appears very unlikely.
Ethics oversight	Human studies were approved by the Ethics Committee of the Medical University of Innsbruck

Note that full information on the approval of the study protocol must also be provided in the manuscript.

Flow Cytometry

Plots

Confirm that:

- [x] The axis labels state the marker and fluorochrome used (e.g. CD4-FITC).
- [x] The axis scales are clearly visible. Include numbers along axes only for bottom left plot of group (a 'group' is an analysis of identical markers).
- [x] All plots are contour plots with outliers or pseudocolor plots.
- [x] A numerical value for number of cells or percentage (with statistics) is provided.

Methodology

Sample preparation	LPO and cell death labelling. Cells derived from cell culture or from IEC isolation procedures were incubated with BODIPY 581/591 C11 or the surface labelling antibodies (see below) at 37°C in the dark for ten to 30 minutes in flow cytometry buffer (2% FCS, 2mM EDTA in PBS). Cells were subsequently washed with PBS, resuspended in FACS buffer and transferred through a 40µm cell strainer for flow cytometry. Annexin V, PI or 7AAD were used for cell death analysis.<br>Mouse LPMCs were isolated according to previously published protocols 60,61. In short, the proximal small intestine was flushed with ice cold PBS, opened longitudinally, cut into small pieces and transferred to HBSS (Gibco, 14175-053) containing 10% FCS, DTT (1mM) and EDTA (2mM) followed by shaking for 20 minutes at room temperature. Samples were vortexed to remove IELs and the tissue was washed and collected in IMDM (Gibco, 21056-023) 20% FCS. Tissue was washed and 10U/ml DNAse (10U/ml, Sigma, D8764) and 128 U/ml collagenase (128U/ml, Sigma, C1889) digested on a shaker for 60 minutes at 37° C. Cells were passed through cell strainers (100µm) and washed twice before transferring to cytometry buffer for staining (see below).<br>Human IEC isolation. The biopsies were moved from RPMI (Biochrome, FG1385) to HBSS-CMF buffer (Gibco, 14175-053, 0.5% BSA, 2mM EDTA and DTT). Samples were incubated on a shaker for 20 minutes at room temperature. The samples were then vortexed vigorously and supernatant (containing IECs) was collected through a 100µm cell strainer, which was repeated for a total of three times. Supernatant was then spun down at 300g and the pellet was used for GPX4 activity assay and western blot analysis as detailed in the respective sections.
Instrument	Gallios Flow Cytometer

Software	Collection of data was done with Gallios Software. Data were analyzed using FlowJo V10
Cell population abundance	No sorting was performed
Gating strategy	For LPO analysis, BODIPY-positive cells among DAPI-negative cells were analyzed as compared to a control sample using BODIPY measurement. For cell death analysis, debris was excluded using FSC/SSC characteristics; Annexin V and PI or 7AAD positivity was determined by flow cytometry.<br>The gating strategy for analyzing the mucosal cellular infiltrate is depicted in Supplementary Figure 9. Briefly, cells were gated using FSC/SSC characteristics. Singlets were selected by comparing FSC width and FSC area. Neutrophils were identified as CD45+, Lin1- (Lin1=CD3, CD19, CD49b, DAPI) and GR1+ cells. Macrophages were identified by CD45+, Lin1- , GR1-, CD11b+, MerTK+. Monocytes were characterized by CD45+, Lin1- , GR1-, Ly6Chi. Dendritic cells were characterized by CD45+, Lin1-, GR1-, CD11c+ and MHCII+. T helper cells were identified by Lin2- (Lin2=CD11c, F4/80, GR1, DAPI), CD3+, CD19-, CD4+. Cytotoxic T cells were identified by Lin2-, CD3+, CD19-, CD8+. B cells were defined as Lin2-, CD3-, CD19+.

☒ Tick this box to confirm that a figure exemplifying the gating strategy is provided in the Supplementary Information.
